# Incorporating prior biological knowledge for network-based differential gene expression analysis using differentially weighted graphical LASSO

**DOI:** 10.1186/s12859-017-1515-1

**Published:** 2017-02-10

**Authors:** Yiming Zuo, Yi Cui, Guoqiang Yu, Ruijiang Li, Habtom W. Ressom

**Affiliations:** 1 Department of Electrical and Computer Engineering, Virginia Polytechnic Institute and State University, Arlington, 22203 VA USA; 2 Department of Radiation Oncology, Stanford University, Palo Alto, 94304 CA USA; 3 Lombardi Comprehensive Cancer Center, Georgetown University, Washington, 20007 DC USA

**Keywords:** Prior biological knowledge, Gaussian graphical model, Weighted graphical LASSO, Network-based differential gene expression analysis

## Abstract

**Background:**

Conventional differential gene expression analysis by methods such as student’s *t*-test, SAM, and Empirical Bayes often searches for statistically significant genes without considering the interactions among them. Network-based approaches provide a natural way to study these interactions and to investigate the rewiring interactions in disease versus control groups. In this paper, we apply weighted graphical LASSO (wgLASSO) algorithm to integrate a data-driven network model with prior biological knowledge (i.e., protein-protein interactions) for biological network inference. We propose a novel differentially weighted graphical LASSO (dwgLASSO) algorithm that builds group-specific networks and perform network-based differential gene expression analysis to select biomarker candidates by considering their topological differences between the groups.

**Results:**

Through simulation, we showed that wgLASSO can achieve better performance in building biologically relevant networks than purely data-driven models (e.g., neighbor selection, graphical LASSO), even when only a moderate level of information is available as prior biological knowledge. We evaluated the performance of dwgLASSO for survival time prediction using two microarray breast cancer datasets previously reported by Bild et al. and van de Vijver et al. Compared with the top 10 significant genes selected by conventional differential gene expression analysis method, the top 10 significant genes selected by dwgLASSO in the dataset from Bild et al. led to a significantly improved survival time prediction in the independent dataset from van de Vijver et al. Among the 10 genes selected by dwgLASSO, UBE2S, SALL2, XBP1 and KIAA0922 have been confirmed by literature survey to be highly relevant in breast cancer biomarker discovery study. Additionally, we tested dwgLASSO on TCGA RNA-seq data acquired from patients with hepatocellular carcinoma (HCC) on tumors samples and their corresponding non-tumorous liver tissues. Improved sensitivity, specificity and area under curve (AUC) were observed when comparing dwgLASSO with conventional differential gene expression analysis method.

**Conclusions:**

The proposed network-based differential gene expression analysis algorithm dwgLASSO can achieve better performance than conventional differential gene expression analysis methods by integrating information at both gene expression and network topology levels. The incorporation of prior biological knowledge can lead to the identification of biologically meaningful genes in cancer biomarker studies.

**Electronic supplementary material:**

The online version of this article (doi:10.1186/s12859-017-1515-1) contains supplementary material, which is available to authorized users.

## Background

Typically, a differential gene expression analysis (e.g., student’s *t*-test, SAM, Empirical Bayes, etc.) is performed to identify genes with significant changes between biologically disparate groups [1–3]. However, independent studies for the same clinical types of patients often lead to different sets of significant genes and had only few in common [4]. This may be attributed to the fact that genes are members of strongly intertwined biological pathways and are highly interactive with each other. Without considering these interactions, differential gene expression analysis will easily yield biased result and lead to a fragmented picture.

Network-based methods provide a natural framework to study the interactions among genes [5]. Data-driven network model reconstructs biological networks solely based on statistical evidence. Relevance network is one common data-driven network model [6, 7]. It uses correlation or mutual information to measure the “relevance” between genes and sets a hard threshold to connect high relevant pairs. Relevance network has extensive application due to its simplicity and easy implementation. However, its drawback becomes significant when the variable number increases: it confounds direct and indirect associations [8]. For example, a strong correlation for gene pair X-Y and X-Z will introduce a less strong but probably still statistically significant correlation for gene pair Y-Z. As a result, when the number of genes is large, relevance network tends to generate over-complicated networks that contain overwhelming false positives. Bayesian network is another classic data-driven network model [9]. Unlike undirected graphs such as relevance networks, Bayesian networks generate directed acyclic graphs, in which each edge indicates a conditional dependence relationship between two genes given their parents. The benefits of using Bayesian networks are: 1) By modeling conditional dependence relationship, Bayesian networks only identify direct associations; 2) With directions in the graph, Bayesian networks allow to infer causal relationship. However, it’s challenging to apply Bayesian networks on high-throughput omic data since learning the structure of Bayesian networks for high dimensional data is time-consuming and can be statistically unreliable. Additionally, Bayesian network cannot model cyclic structures, such as feedback loops, which are common in biological networks.

Recently, Gaussian graphical models (GGMs) have been increasingly applied on biological network inference [10–12]. Similar to Bayesian network, GGMs can remove the effect of indirect associations through estimation of the conditional dependence relationship. At the same time, they generate undirected graphs and have no limitation on modeling only acyclic structures. In GGMs, a connection between two nodes corresponds to a non-zero entry in the inverse covariance matrix (i.e., precision matrix), which indicates a conditional dependency between these two nodes given the others. GGMs dates back to early 1970s when Dempster introduced “covariance selection” problem [13]. The conventional approach to solve this problem relies on statistical test (e.g., deviation tests) and forward/backward selection procedure [14]. This is not feasible for high-throughput omic data when the number of genes is ranging from several hundred to thousands while the number of samples are only tens to hundreds. In addition, the “small *n*, large *p*” scenario for omic data (i.e., sample size is far less than the variable number), makes maximum likelihood estimation (MLE) of precision matrix not to exist because the sample covariance matrix is rank deficient. To deal with these issues, Schäfer et al. proposed to combine Moore-Penrose pseudoinverse and bootstrapping technique to approximate the precision matrix [15]. Others applied *ℓ*_1_ regularization to get a sparse network [16–18]. Taking into account of the sparsity property of biological networks and the computational burden of bootstrapping, *ℓ*_1_ regularization methods are preferred. Among various *ℓ*_1_ regularization methods, Meinshausen et al. performed *ℓ*_1_ regularized linear regression (i.e., LASSO) for each node to select its “neighbors” [16]. Given all its neighbors, one node is conditionally independent with the remaining ones. Since LASSO is performed for each node, this ‘neighbor selection’ approach may face a consistency problem. For example, while gene X is selected as Y’s neighbor, gene Y may not be selected as X’s neighbor when performing LASSO for gene X and gene Y separately. Compared with neighbor selection method, a more reasonable approach is graphical LASSO, which directly estimates precision matrix by applying *ℓ*_1_ regulation on the elements of the precision matrix to obtain a sparse estimated precision matrix [17, 18]. We will pursuit the extension of graphical LASSO in this paper.

In additional to data-driven network models, there are many publicly available databases such as STRING (http://string-db.org), KEGG (http://www.genome.jp/kegg), BioGRID(http://thebiogrid.org/), and ConsensusPathDB (http://consensuspathdb.org/), where one can extract various types of interactions including protein-protein, signaling, and gene regulatory interactions [19–22]. Biological networks reconstructed from these databases have been reported useful. For example, Chuang et al. reconstructed protein-protein interaction (PPI) network from multiple databases to help identify markers of metastasis for breast cancer studies using gene expression data [23]. They overlaid the gene expression value on its corresponding protein in the network and searched for sub-networks whose activities across all patients were highly discriminative of metastasis. By doing this, they found several hub genes related to known breast cancer mutations, while these genes were not found significant by conventional differential gene expression analysis. They also reported that the identified sub-networks are more reproducible between different breast cancer cohorts than individual gene markers. However, databases are far from being complete. Networks constructed purely based on the databases have a large number of false negatives. In addition, databases are seldom specific to a certain disease, so the interactions that exist in the databases may not be reflective of the patient population under study. In contrast, data-driven models are likely to have a large number of false positives due to background noise. Considering this, an appropriate approach to integrate the prior biological knowledge from databases and data-driven network model is desirable for more robust and biologically relevant network reconstruction [24].

**Fig. 1:**
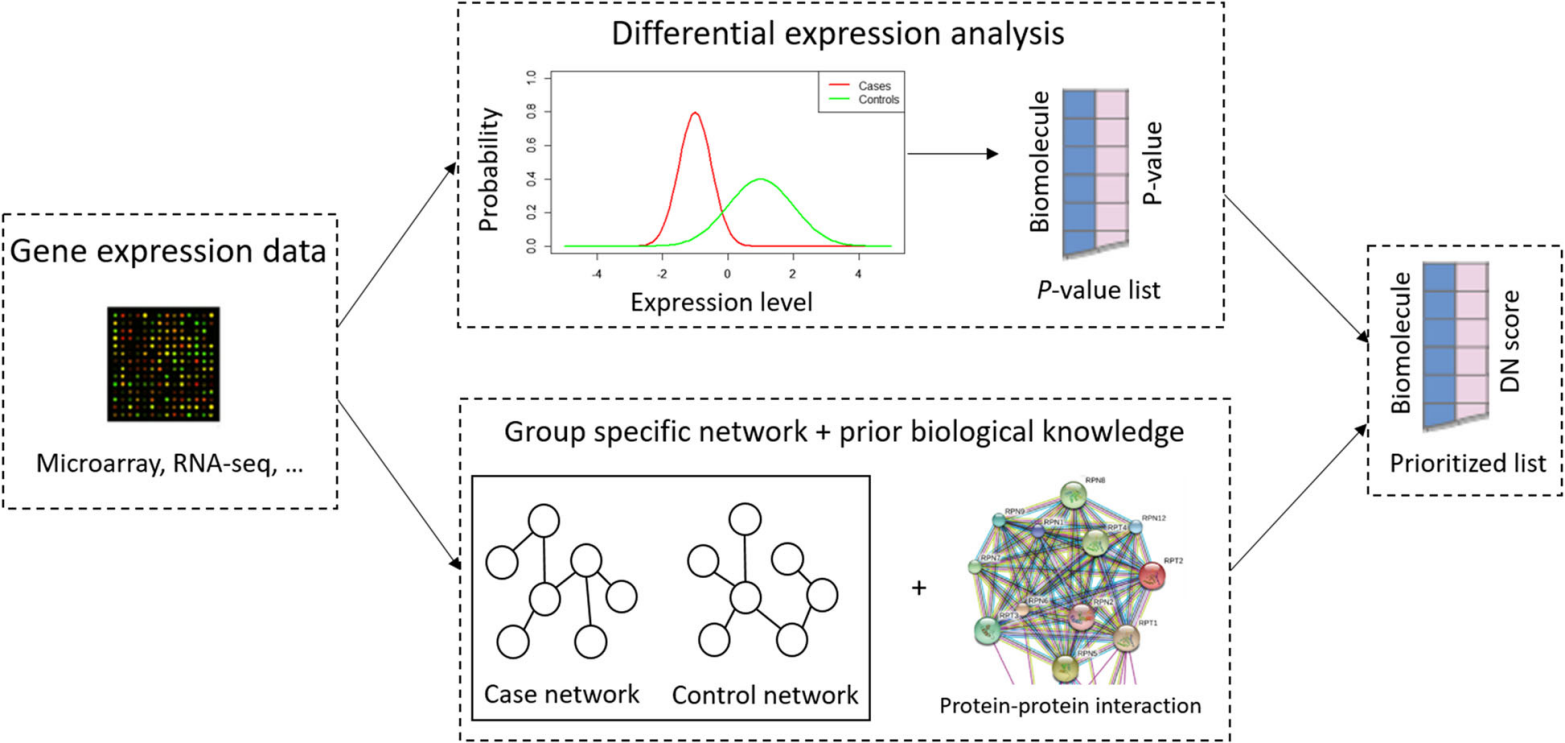
An overview of dwgLASSO. The input is gene expression data (e.g., Microarray, RNA-seq data, etc.) and the output is a prioritized list based on the differential network (DN) score defined within dwgLASSO

Previously, prior biological knowledge has been incorporated into the neighbor selection method [25]. It relies on the Bayesian interpretation of LASSO and assigns two different prior distributions for connections that are present in the database and those are not. Recently, weighted graphical LASSO (wgLASSO) has been proposed to incorporate prior biological knowledge into graphical LASSO by assigning different weights to the entries of precision matrix [26]. In this work, we extend the original wgLASSO algorithm, explain this idea from a Bayesian perspective, and perform comprehensive comparisons between wgLASSO and competing data-driven network models (e.g., neighbor selection, graphical LASSO). Additionally, exploring the topological changes between biological disparate groups may lead to new discoveries that cannot be identified by conventional differential gene expression analysis [27–29]. For example, high-degree nodes (i.e., hubs) that only exist in one of the biologically disparate groups may indicate the regulatory rule of the hub genes only in that group. Knowledge-fused differential dependency network (KDDN) is a recently proposed method to construct knowledge incorporated network that can show the rewiring connections between two groups [29]. An open-source Cytoscape app is available for easy implementation [30]. In this paper, we propose a novel algorithm called differentially weighted graphical LASSO (dwgLASSO) for network-based differential gene expression analysis. This is achieved by building separate networks for biologically disparate groups using wgLASSO, exploring the topological changes between different groups, and prioritizing significant gene list from conventional differential gene expression analysis as shown in Fig. 1. Other previously reported methods include those that focus on integrating prior biological knowledge into data-driven network model to identify sub-networks that are related to the disease under study [31, 32]. Our work differs with these methods since we compute a differential network score for each gene and prioritize them for subsequent analysis rather than outputting a sub-network list for biological interpretation. Also, methods that directly incorporate gene networks or prior biological knowledge into statistical models for classification and regression tasks have been reported [33, 34]. The rationale is that functionally linked genes tend to be co-regulated and co-expressed, and therefore should be treated similarly in the statistical model. Our work leaves the statistical model untouched. Instead, it focuses on using the best set of gene biomarkers as an input to the statistical model. This is considered to have advantages over providing multiple linked genes from the network whose expression values have similar patterns. We show the application of dwgLASSO on two independent microarray datasets from breast cancer patients for survival time prediction, and on TCGA RNA-seq data acquired from patients with hepatocellular carcinoma (HCC) for classification task between tumor samples and their corresponding non-tumorous liver tissues.

The rest of the paper is organized as follows. “Methods” section introduces the extended wgLASSO algorithm and the proposed dwgLASSO for network-based differential gene expression analysis. “Results and discussion” section presents the results of wgLASSO and dwgLASSO based on simulation, microarray and RNA-seq data. Finally, “Conclusion” section summarizes our work and discusses possible future extensions.

## Methods

### Network inference using wgLASSO

Consider a centered and scaled data matrix $\mathbf {X}_{n\times p} \left (\mathrm {i.e.}, \sum _{i=1}^{n} x_{ij}=0, \sum _{i=1}^{n} x_{ij}^{2}=1\right)$, it measures the intensities of *p* genes on *n* samples, from a *p*-dimensional Gaussian distribution with zero means on each dimension and positive definite covariance matrix **Σ**_*p*×*p*_ (i.e., $\mathbf {X} \sim \mathcal {N}(\mathbf {0},\mathbf {\Sigma })$). Suppose the sample size *n* is far less than the variable number *p* (i.e., *n*≪*p*), then the MLE of the precision matrix (i.e., ***Θ***=**Σ** ^−1^) does not exist since the sample covariance matrix **S** is rank deficient. If we further assume ***Θ*** is sparse, then a *ℓ*_1_ regularization term can be added to the negative log-likelihood function *f*(**X**|***Θ***)=− log det***Θ***+tr(**S** ***Θ***) for a sparse precision matrix estimation as shown in Eq. (1). Graphical LASSO is an algorithm to efficiently solve Eq. (1) by using block coordinate descent [8, 9]. Once the sparse precision matrix $\hat {\boldsymbol {\Theta }}$ is obtained, a non-zero element in $\hat {\boldsymbol {\Theta }}$ (i.e., $\hat {\theta }_{ij}\neq 0$) indicates a conditional dependence between **x**_*i*_ and **x**_*j*_ given the others. For network $\mathcal {G}=\{(i,j);1\le i<j\le p\}$, we have $\hat {\mathcal {G}}=\{(i,j):\hat {\theta }_{ij}\neq 0\}$.

1 $$ \underset{\boldsymbol{\Theta}\succ\mathbf{0}}{\text{arg min}} -\log\det\boldsymbol{\Theta}+\text{tr}(\mathbf{S}\boldsymbol{\Theta})+\lambda\left\lVert{\boldsymbol{\Theta}}\right\rVert_{1}  $$

where ***Θ*** is the precision matrix, ***Θ***≻**0** is the constraint that ***Θ*** has to be positive definite, **S** is the sample covariance matrix, tr denotes the trace, the sum of the diagonal elements in a matrix, ‖***Θ***‖_1_ represents the *ℓ*_1_ norm of ***Θ***, the sum of the absolute values of all the elements in ***Θ***, and *λ* is the tuning parameter controlling the sparsity of ***Θ***.

LASSO based estimates have a Bayesian interpretation [35]. $\hat {\boldsymbol {\Theta }}$ is the maximum a posteriori (MAP) estimate for the posterior distribution *p*(***Θ***|**X**) with a Laplacian prior distribution *p*(***Θ***) as shown in Eq. (2). The LASSO term *λ*‖***Θ***‖_1_ in Eq. (1) is now part of *p*(***Θ***)= exp(−*λ*‖***Θ***‖_1_) with zero means and a scaling parameter *λ*. From the Bayesian perspective, *p*(***Θ***) encodes the prior knowledge of the network topology. For a database that contains only binary information (connecting or not) for a given gene pair, a natural way is to assign two different scaling parameters *λ*_1_ and *λ*_2_ for connecting pairs and those are not connected, as shown in Eq. (3). For connecting pairs, their Laplacian prior distribution is diffused, while for non-connecting pairs their Laplacian prior distribution is concentrated (i.e., *λ*_1_≫*λ*_2_). In another word, a larger penalty will be assigned to non-connecting pairs to increase the chance of their corresponding entries in ***Θ*** to shrink to zero. In reality, tuning *λ*_1_ and *λ*_2_ at the same time involves two dimensional grid search, which is quite time-consuming for high-dimensional data. An extreme solution to set *λ*_2_=0 links all the connecting gene pairs from the database in the graph, neglecting the fact that the database might contain some spurious connections for the disease under study.

2 $$\begin{array}{@{}rcl@{}}{} p(\boldsymbol{\Theta}|\mathbf{X}) &=&\!\frac{p(\mathbf{X}|\boldsymbol{\Theta})p(\boldsymbol{\Theta})}{p(\mathbf{X})} \\ &&\propto \!\exp(\log\det\boldsymbol{\Theta}-\text{tr}(\mathbf{S}\boldsymbol{\Theta}\!))\!\times \!\exp(-\lambda\left\lVert{\boldsymbol{\Theta}}\right\rVert_{1}\!) \end{array} $$

3 $$\begin{array}{@{}rcl@{}} p(\boldsymbol{\Theta}) &=\!& \exp(-\lambda_{1}\!\sum\!\left\lVert{\boldsymbol{\Theta}_{non-con}}\right\rVert_{1}\!)\,-\,\lambda_{2}\!\sum \left\lVert{\boldsymbol{\Theta}_{con}}\right\rVert_{1}\!) \end{array} $$

Instead of using the binary information, a continuous confidence score is more suitable to incorporate prior biological knowledge into graphical LASSO. The confidence score can be obtained from multiple resources. For example, an estimated functional association score for PPIs is provided by STRING database. We scale this confidence score into the range [0,1] and create a weight matrix **W**_*p*×*p*_. In **W**, 1 indicates a complete trust for a gene pair to be connected, 0 represents that no evidence supports a gene pair to be connected. In this way, we can assign different penalties to different gene pairs as shown in Eq. (4). Compared to Eq. (3), (4) also gives larger penalty for less likely connecting gene pairs, but now there is only one tuning parameter *λ*. For a fixed *λ*, R package glasso can solve Eq. (4) efficiently given **W** [17].

4 $$ \underset{\boldsymbol{\Theta}\succ\mathbf{0}}{\text{arg min}} -\log\det\boldsymbol{\Theta}+\text{tr}(\mathbf{S}\boldsymbol{\Theta})+\lambda\left\lVert{(\mathbf{1}-\mathbf{W})*\boldsymbol{\Theta}}\right\rVert_{1}  $$

where **1** is all 1 matrix, **W** is the weight matrix containing the confidence score for each gene pair and ∗ represents the element-wise multiplication between two matrices.

For LASSO based optimization problem as shown in Eq. (4), tuning the parameter *λ* is crucial since it controls the sparsity of the output $\hat {\boldsymbol {\Theta }}$. Typically, *λ* is tuned by cross-validation, Akaike information criterion (AIC), Bayesian information criterion (BIC), or stability selection [36]. Considering that AIC and BIC often lead to data under-fitting (i.e., over-sparse network) and stability selection requires extensive computational time, we prefer to use cross validation with one standard error rule to select the optimal tuning parameter *λ* ^*opt*^. By using one standard error rule, we can achieve the simplest (most regularized) model whose error is within one standard deviation of the minimal error. Our wgLASSO algorithm is shown below.



### Network-based differential gene expression analysis using dwgLASSO

**Fig. 2:**
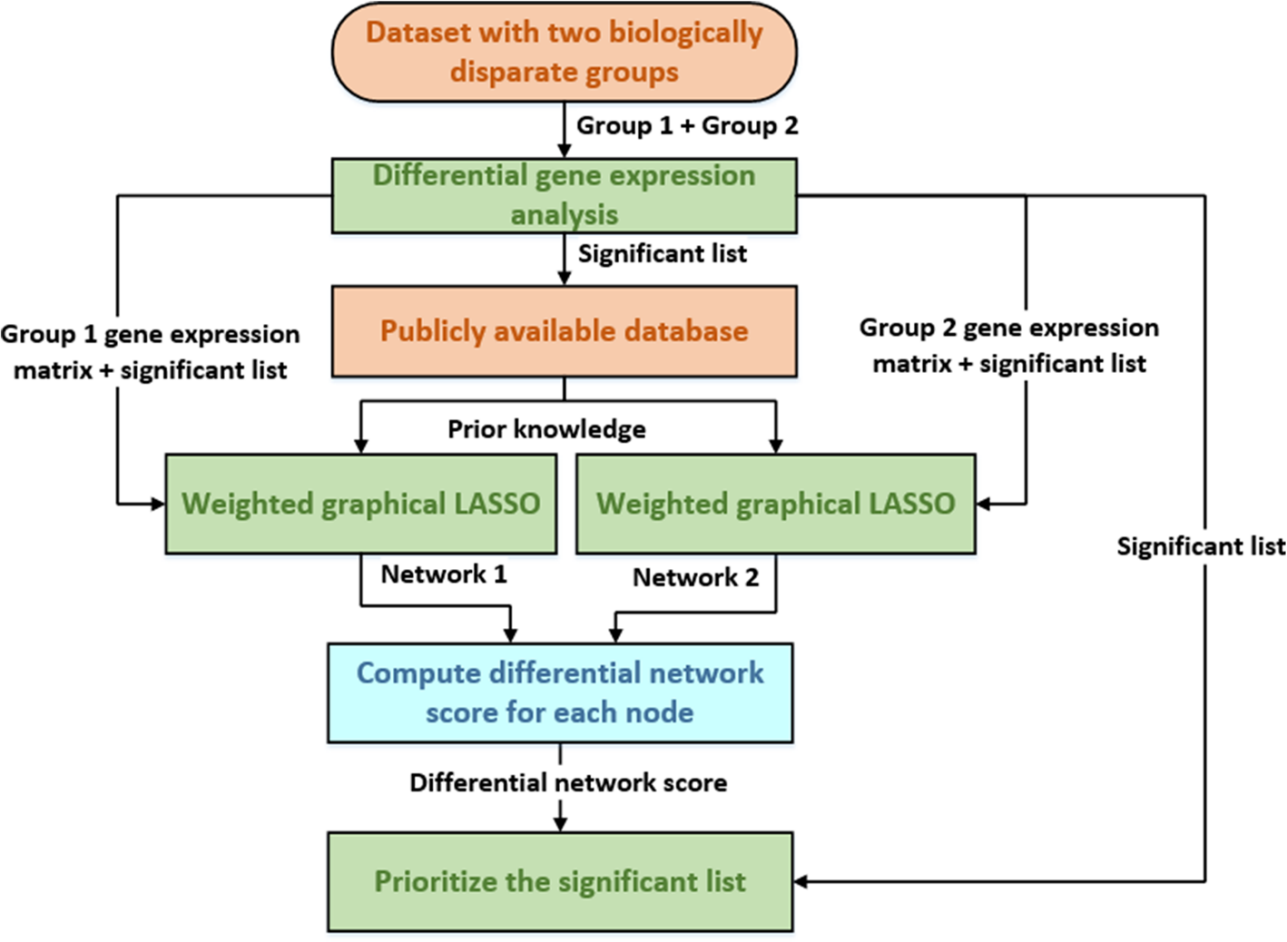
Framework for dwgLASSO



Figure 2 shows the framework of the proposed dwgLASSO algorithm for network-based differential gene expression analysis. dwgLASSO prioritizes the significant list obtained from the conventional differential gene expression analysis based on the topological changes between the group-specific networks built by wgLASSO. Specifically, dwgLASSO first performs differential gene expression analysis to obtain a list of significant genes whose expression values differ between the two biologically disparate groups. Then based on these significant genes, dwgLASSO builds group specific networks using wgLASSO. After the networks are constructed, dwgLASSO calculates a differential network score for each gene in the significant list based on the topological changes between the two group-specific networks. In calculating the differential network score, dwgLASSO first computes the node degree for each gene in both networks, meaning the number of neighbors each gene is connected with. Then considering the size of the two networks are different, the node degrees are scaled into the range [0,1]. At last, the differential network score for one gene is computed as the absolute value of the difference between the two associated scaled node degrees from different groups. Finally, with the differential network scores, dwgLASSO prioritizes the significant list from the conventional differential gene expression analysis in a decreasing order. The prioritized gene list is used for subsequent analysis such as building classification or regression models. We believe dwgLASSO can help classification or regression models to achieve better prediction performance since the prioritized list integrates information at the gene expression and network structure levels. More than that, the incorporation of prior biological knowledge is more likely to identify biologically meaningful genes. Detailed algorithm for dwgLASSO is shown below.

## Results and discussion

### Simulation data

Biological networks are reported to be scale-free, which means the degree distribution of the network follows a power law [37]. We considered this scale-free property of biological network in generating simulation data using R package huge [38]. Using huge, a scale-free network was built by inputting the node number *p*. The sparsity of the network *s* is fixed, depending on *p*. For example, when the node number is 100, the sparsity of the network is 0.02, indicating only 2% of all possible connections (i.e., $\frac {p\times (p-1)}{2}$) exist in the scale-free network. Once the scale-free network is built, huge creates the true precision matrix ***Θ***_*true*_ based on the network topology and the positive definite constraint ***Θ***_*true*_≻**0** so that **Σ**_*true*_=(***Θ***_*true*_)^−1^ exists. At last, simulation data $\mathbf {X}_{n\times p} \sim \mathcal {N}(\mathbf {0},\mathbf {\Sigma }_{true})$ was generated.

We created simulation datasets with various *p* and *n*, as seen in Table 1. The weight matrix **W**, which contains prior biological knowledge, was constructed based on ***Θ***_*true*_. In reality, databases may also contain spurious connections for the disease under study. To evaluate how the incorrect connections in **W** will impact wgLASSO, we introduced an additional metric, *acc*. When *a* *c* *c*=60*%*, we randomly reassigned 40% incorrect connections in **W**. Specifically, **W** was created as follows. Initially, for zero entries in ***Θ***_*true*_, the corresponding entries in **W** were also zero; for non-zero entries in ***Θ***_*true*_, the corresponding entries in **W** were randomly generated from the uniform distribution $\mathcal {U}(0,1)$. Then, we randomly assigned incorrect connections into **W** based on the *acc* value while keeping the total connections in **W** the same as those in ***Θ***_*true*_. Under the assumption that incorrect entries in **W** should have lower confidence scores compared to those of correct entries, we generated incorrect entries from the uniform distribution $\mathcal {U}(0,0.5)$.

**Table 1:** The mean and standard deviation (in parenthesis) of false positives (FP) and false negatives (FN) for connections from neighbor selection (NS), graphical LASSO (gLASSO) and weighted graphical LASSO (wgLASSO) methods under different node number (*p*) and sample size (*n*) scenarios

*p*	*n*	NS (or)		NS (and)		gLASSO		wgLASSO (*a* *c* *c*=60*%*)		wgLASSO (*a* *c* *c*=40*%*)	
		FP	FN	FP	FN	FP	FN	FP	FN	FP	FN
100	50	150 (17)	151 (10)	166 (15)	157 (10)	154 (23)	148 (11)	**1** **1** **2** **(** **1** **7** **)**	**1** **0** **4** **(** **1** **1** **)**	129 (18)	122 (11)
	100	113 (16)	111 (15)	132 (17)	122 (16)	114 (20)	112 (15)	**8** **2** **(** **1** **5** **)**	**7** **4** **(** **1** **3** **)**	93 (16)	87 (12)
	200	69 (13)	59 (18)	78 (15)	72 (21)	79 (17)	63 (19)	**5** **1** **(** **1** **1** **)**	**3** **9** **(** **1** **4** **)**	58 (13)	50 (15)
500	250	707 (42)	679 (77)	758 (43)	738 (82)	710 (48)	681 (77)	**4** **8** **0** **(** **3** **6** **)**	**4** **5** **1** **(** **6** **6** **)**	549 (39)	526 (60)
	500	425 (30)	453 (129)	473 (42)	493 (134)	431 (40)	468 (129)	**2** **7** **7** **(** **2** **6** **)**	**2** **9** **0** **(** **8** **7** **)**	330 (31)	313 (106)
	1000	175 (22)	164 (117)	189 (27)	177 (118)	199 (28)	186 (126)	**1** **0** **9** **(** **1** **8** **)**	**1** **1** **0** **(** **7** **6** **)**	130 (21)	135 (88)

The best performance is marked in bold

We estimated the true network topology by using neighbor selection, graphical LASSO, and the proposed wgLASSO methods. For neighbor selection method, two strategies were applied to deal with the inconsistency problem. Neighbor selection with “or” operator accepted inconsistent connections while neighbor selection with “and” operator rejected them. To make a fair comparison, we tuned the regularization parameter in each method to ensure the output network has the same sparsity as the true network (i.e., *s*=0.02 for *p*=100, *s*=0.004 for *p*=500). For each *n* and *p* scenario, we regenerated **X**_*n*×*p*_ 100 times, calculated the false positives and false negatives of connections for each method, and listed their means and standard deviations in Table 1. To evaluate how the incorrect connections in **W** would impact the performance of wgLASSO, we randomly reassigned 40% (*a* *c* *c*=60*%*) and 60% (*a* *c* *c*=40*%*) incorrect prior biological knowledge in **W**. From Table 1, we can conclude that the estimated network from wgLASSO has much less false positives and false negatives, compared with those from neighbor selection and graphical LASSO methods. A decrease of *acc* in **W** would lead to more false positives and false negatives from wgLASSO, but it still outperforms neighbor selection and graphical LASSO methods when the *acc* in **W** is only as moderate as 40%.

**Fig. 3:**
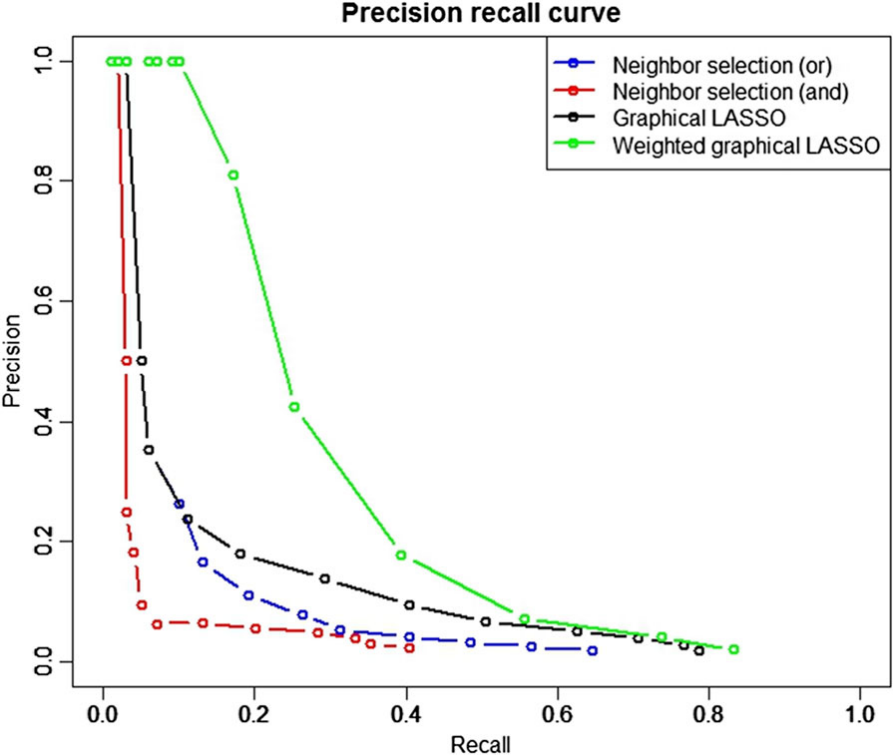
Precision recall curves for neighbor selection, graphical LASSO and weighted graphical LASSO methods under *p*=100,*n*=50 and *a* *c* *c*=40*%*

To make more comprehensive comparison, we plotted precision recall curve to evaluate the performance of neighbor selection, graphical LASSO and wgLASSO methods. We ran the above methods with *p*=100,*n*=50 and *a* *c* *c*=40*%* in **W**, computed the precision and recall, and generated the plot as shown in Fig. 3. From Fig. 3, wgLASSO displays a clear improvement over neighbor selection and graphical LASSO methods. This agrees with our expectation since wgLASSO considers whether the connection has supporting evidence from database and how well it fits the data in the model.

### Microarray data

We applied the proposed dwgLASSO algorithm on two breast cancer microarray datasets: Bild et al. and van de Vijver et al. datasets [39, 40]. The former includes 158 patients with all their survival records, and was used for training. We excluded patients with less than 5-year follow-up time. Among the remaining patients, 42 with less than 5-year survival during the follow-up time were considered to form high risk group while the other 60 formed the low risk group. van de Vijver et al. dataset contains 295 breast cancer patients, together with their survival records, and was used for independent testing. Both datasets are available at PRECOG website (https://precog.stanford.edu), an online repository for querying cancer gene expression and clinical data, and have been preprocessed for subsequent statistical analysis [41]. The raw Bild et al. and van de Vijver et al. datasets are also available at Gene Expression Omnibus (GSE3143) and R package seventyGeneData, respectively [42].

**Fig. 4:**
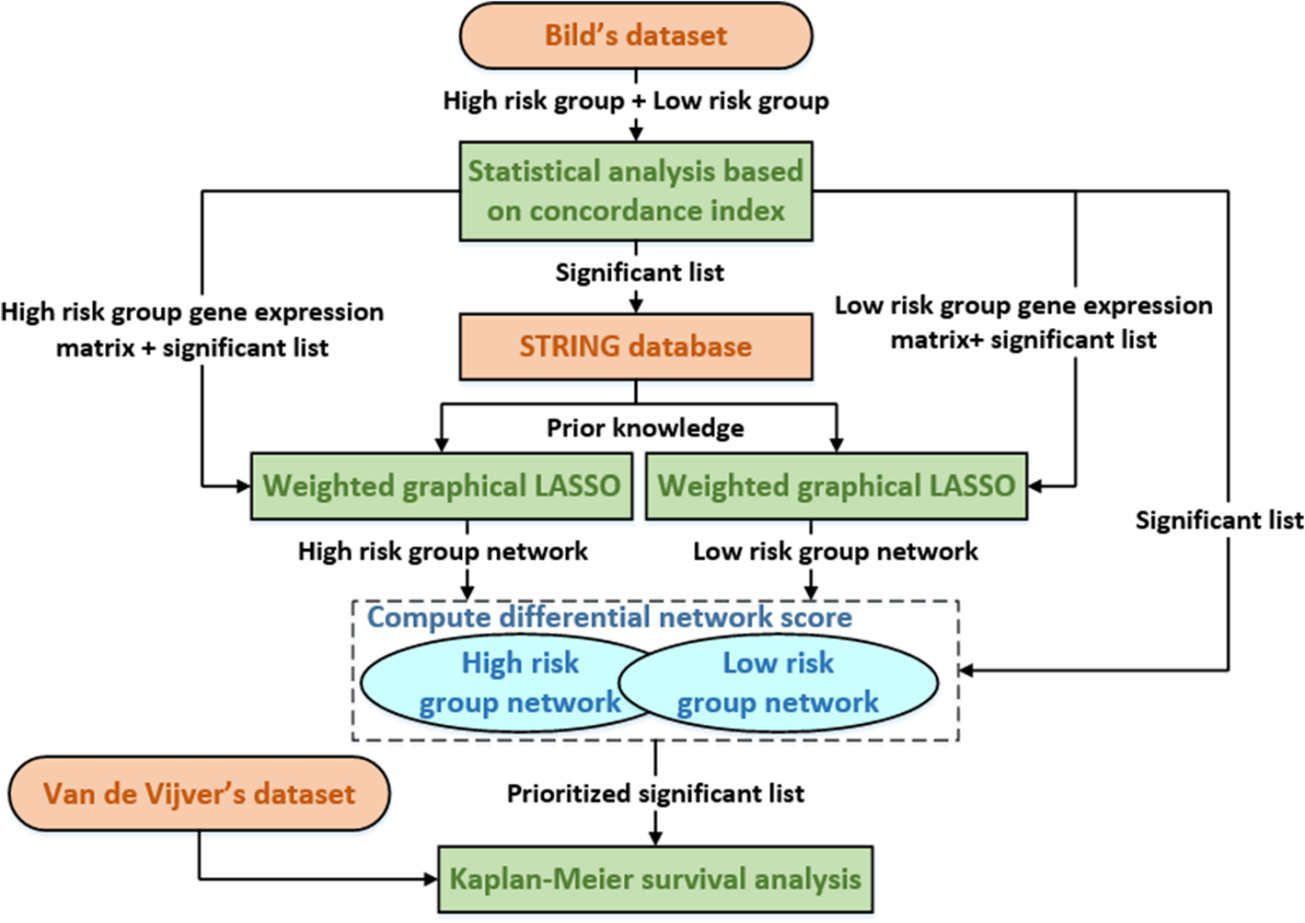
Workflow of dwgLASSO for more accurate survival time prediction on microarray data

**Fig. 5:**
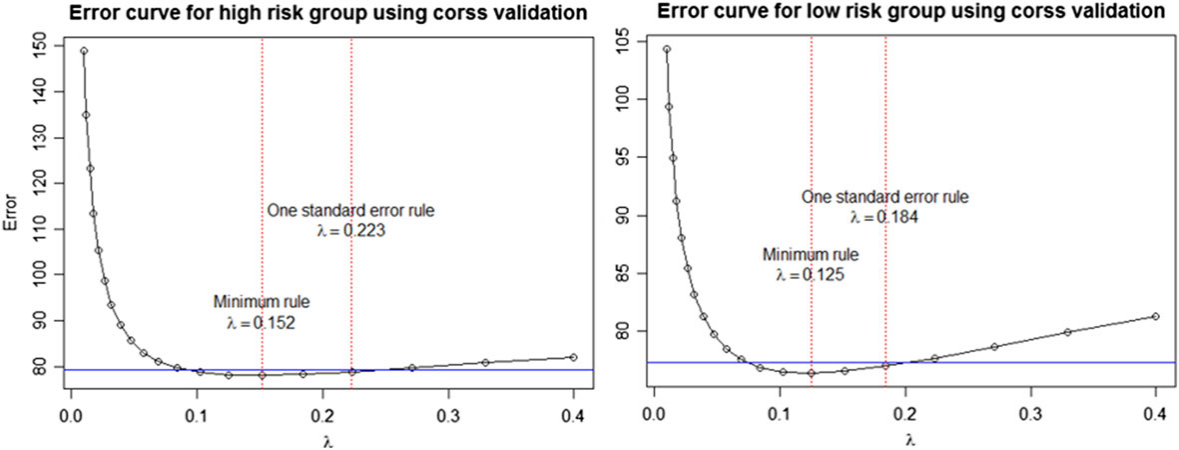
Error curves to choose optimal tuning parameter *λ* ^*opt*^ using 10-fold cross validation by one standard error rule. The *blue* line indicates the one standard error for *λ* ^*min*^ in the direction of increasing regularization

Our interest is to obtain a prioritized significant gene list based on dwgLASSO for more accurate survival time prediction. The workflow is shown in Fig. 4. We first performed univariate analysis on Bild et al. dataset to select a list of statistically significant genes based on concordance index between the expression value and survival time [43]. This lead to a total of 58 genes whose adjusted *p*-values were less than 0.05. The inflation of Type I error caused by multiple testing was controlled by the false discovery rate (FDR) using the Benjamini-Hochberg procedure. The total 58 significant genes are included in Additional file 1: Table S1 along with their associated adjusted *p*-values. We then applied wgLASSO algorithm to build two separate networks using the total 58 significant genes for the high risk and low risk groups, respectively. The weight matrix **W** was constructed based on the confidence scores from STRING database after inputting the 58 significant genes to investigate the PPIs among them. For gene pairs with no confidence scores from STRING, we assigned the corresponding entries in **W** to zeros. In wgLASSO, we performed 10-fold cross validation and chose the optimal tuning parameter *λ* ^*opt*^ by one standard error rule. Fig. 5 shows our chose of *λ* ^*opt*^: *λ* ^*opt*^=0.223 for high risk group and *λ* ^*opt*^=0.184 for low risk group. From the networks, we calculated the node degree for each gene in two groups $\left (d_{i}^{h}, d_{i}^{l}\right)$, scaled them based on the network size $\left (sd_{i}^{h}, sd_{i}^{l}\right)$, and computed the differential network score $\left (dns_{i}=|sd_{i}^{h}-sd_{i}^{l}|\right)$. At last, we prioritized the 58 significant genes based on the network differential scores in a decreasing order.

**Fig. 6:**
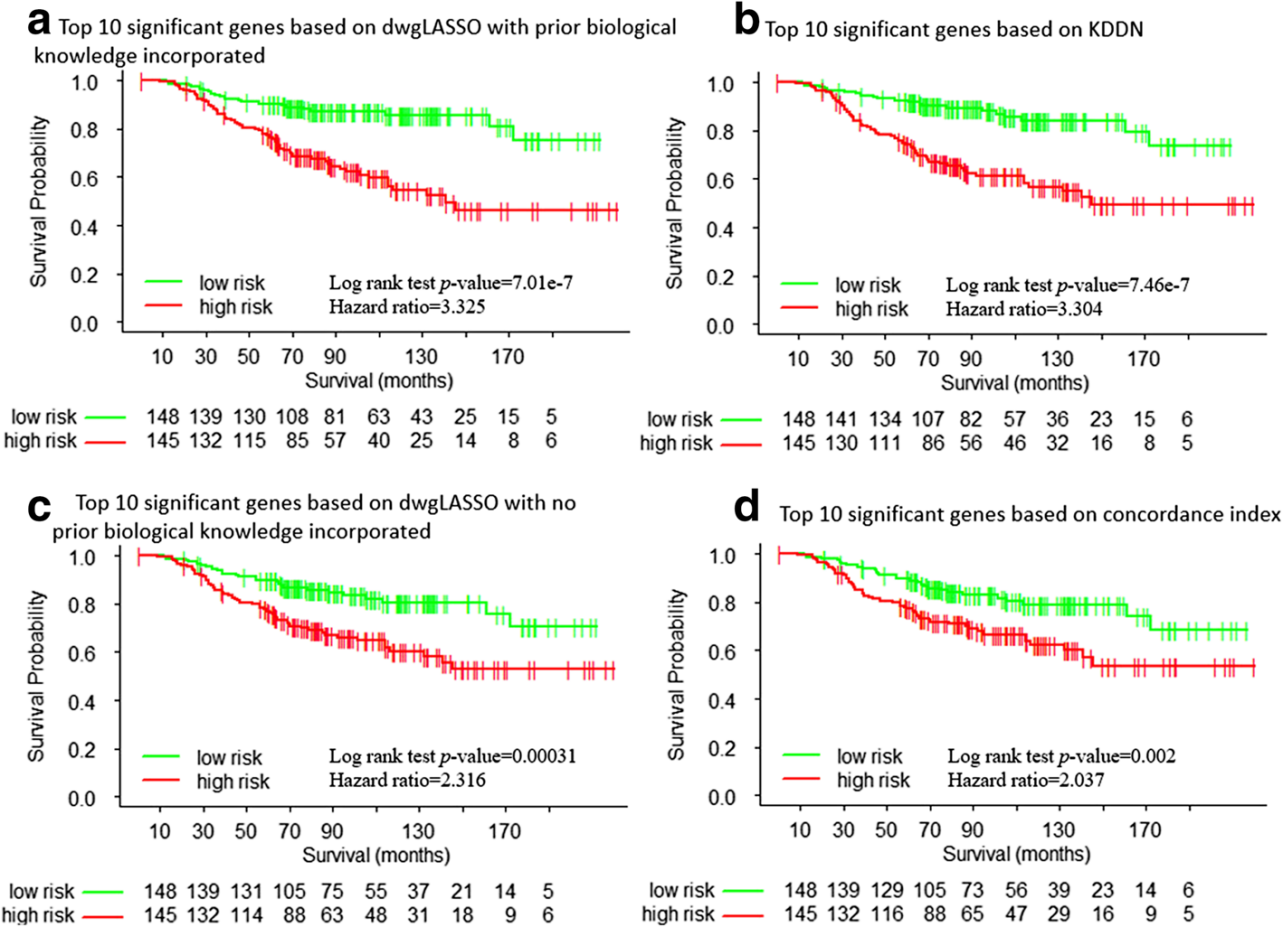
Survival curves. **a** top 10 significant genes based on dwgLASSO with prior biological knowledge incorporated, **b** top 10 significant genes based on KDDN, **c** top 10 significant genes based on dwgLASSO with no prior knowledge incorporated, **d** top 10 significant genes based on concordance index

To evaluate whether dwgLASSO could lead to more accurate survival time prediction, we tested the prioritized gene list using different methods on the independent van de Vijver et al. dataset. The 295 patients were divided into high risk and low risk groups according to the risk scores calculated using multivariate Cox regression from the top 10 significant genes based on dwgLASSO, a competing prior knowledge incorporated network analysis method (i.e., KDDN), and conventional differential gene expression analysis (i.e., concordance index). Unlike dwgLASSO that builds group-specific networks, KDDN generates only one network with all rewiring connections. From the network constructed by KDDN, we computed the node degree for each gene to help prioritize the significant gene list. Kaplan-Meier survival analysis was then performed to evaluate the performance of the above three scenarios. The resulting survival curves are shown in Figs. 6 a, b, and d. To evaluate how much the incorporation of prior biological knowledge contributes to the improved performance of dwgLASSO, we tested the top 10 significant genes selected based on dwgLASSO with no prior biological knowledge incorporated (i.e., **W**=**0**). The resulting survival curve is shown in Fig. 6 c. As expected, dwgLASSO with no prior biological knowledge incorporated is equivalent to using graphical LASSO in building group specific networks (Fig. 4). As illustrated in Fig. 6, the top 10 significant genes from dwgLASSO with prior biological knowledge incorporated yielded the best performance (*p*−value=7.01×10^−7^, hazard ratio =3.325), compared to the top 10 significant genes from KDDN (*p*−value=7.46×10^−7^, hazard ratio =3.304), the top 10 significant genes based on dwgLASSO with no prior biological knowledge incorporated (*p*−value=0.00031, hazard ratio =2.316), and the top 10 significant genes based on concordance index (*p*−value=0.002, hazard ratio =2.037). We believe the improved performance achieved by dwgLASSO and KDDN are due to the extra information provided from the topological changes between high risk and low risk groups. Also, dwgLASSO and KDDN benefit from incorporating prior biological knowledge to obtain more reliable and biologically relevant genes shared across independent datasets, leading to better prediction performance than those that do not use prior biological knowledge (Fig. 6). Table 2 presents the top 10 significant genes selected based on concordance index and dwgLASSO with prior biological knowledge incorporated, together with their adjusted *p*-values. The top 10 genes from the other methods are presented in Additional files 2: Table S2.

**Table 2:** The top 10 significant genes based on conventional differential gene expression analysis (i.e., concordance index) and dwgLASSO with prior biological knowledge incorporated, along with their adjusted *p*-value

Top 10 significant genes based on concordance index		Top 10 significant genes based on dwgLASSO	
Gene symbol	Adjusted *p*-value	Gene symbol	Adjusted *p*-value
BTD	0.000167029	SALL2	0.018149333
FKTN	0.000424976	UBE2S	0.015577505
LRRC17	0.000424976	**R** **A** **B** **1** **1** **F** **I** **P** **5**	0.001638818
**R** **A** **B** **1** **1** **F** **I** **P** **5**	0.001638818	KIAA1467	0.005012636
**E** **M** **X** **2**	0.002384716	XBP1	0.005019825
HNRNPAB	0.002384716	KIAA0922	0.021163875
TKT	0.002805234	**E** **M** **X** **2**	0.002384716
LANCL1	0.003481701	OAZ2	0.040090787
TFF3	0.003481701	NDC80	0.030630047
USF2	0.004094746	CCT5	0.048116117

Common genes are marked in bold

Among the top 10 significant genes based on dwgLASSO in Table 2, UBE2S has been reported to be over-expressed in breast cancer [44]. The authors showed UBE2S knockdown suppressed the malignant characteristics of breast cancer cells, such as migration, invasion, and anchorage-independent growth. SALL2 has also been reported as a predictor of lymph node metastasis in breast cancer [45]. Unlike UBE2S, SALL2 was identified as a tumor suppressor gene that can suppress cell growth when over-expressed [46]. Additionally, XBP1 has been reported to be activated in triple-negative breast cancer and has a pivotal role in the tumorigenicity and progression of this breast cancer subtype [47]. KIAA0922 has also been reported as a novel inhibitor of Wnt signaling pathway, which is closely related to breast cancer [48]. None of UBE2S, SALL2, XBP1 and KIAA0922 is among the top 10 significant genes based on concordance index according to Table 2.

**Fig. 7:**
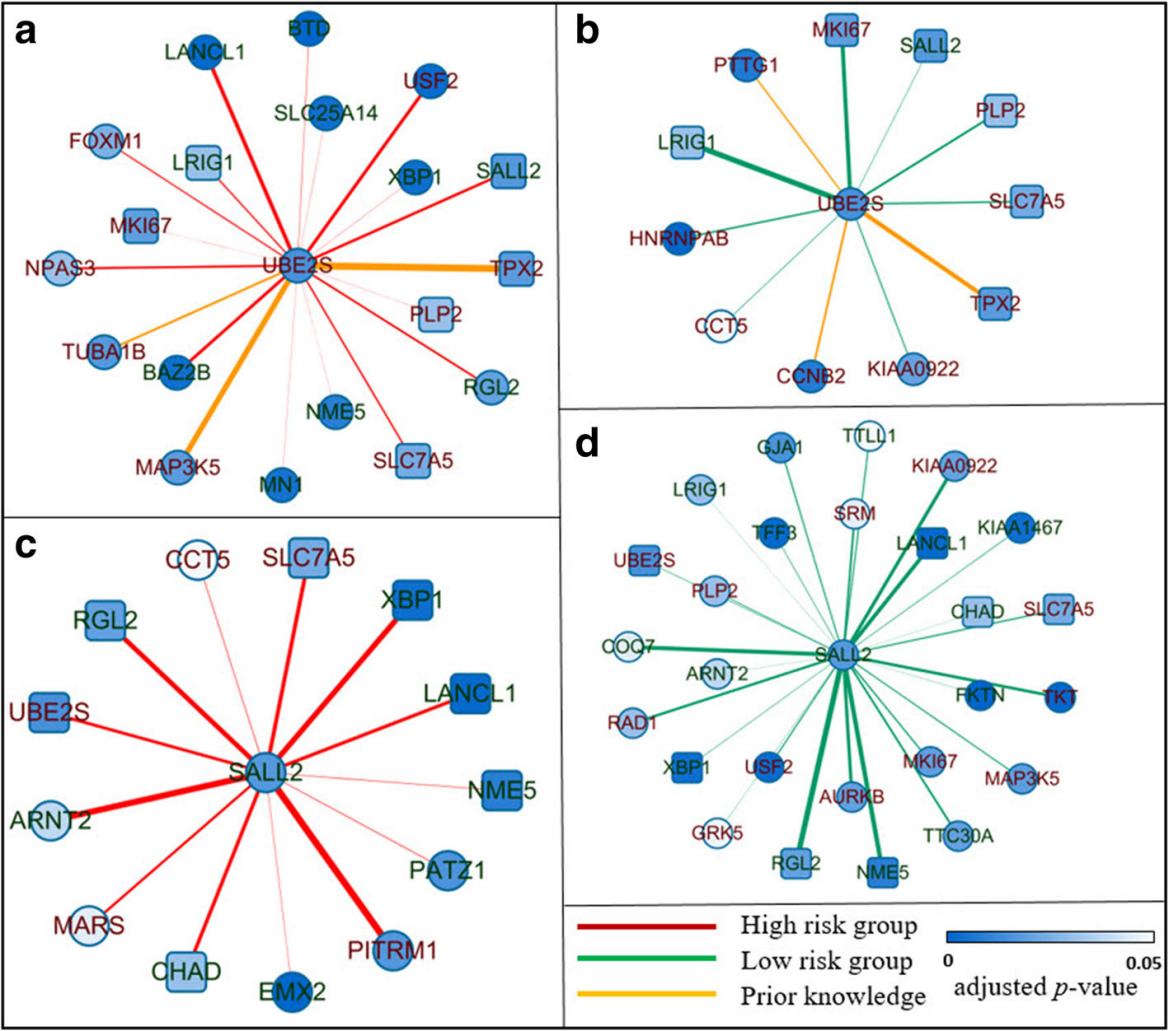
Neighbors of UBE2S and SALL2 in two groups. **a** neighbors of UBE2S in the high risk group, **b** neighbors of UBE2S in the low risk group, **c** neighbors of SALL2 in the high risk group, **d** neighbors of SALL2 in the low risk group. Label colors represent *over- (red)* or *under- (green)* expression in the high risk group. Node shapes indicate unique (*circle*) or shared (*rectangle*) genes between the two groups. Node colors show the significance of the gene expression value between the two groups. *Yellow edges* represent interactions recorded in the STRING database. Thickness of the edge indicates the strength of the interaction

In Fig. 7, we showed the neighbors of UBE2S and SALL2 in the high risk and low risk groups based on the networks created by wgLASSO from Bild et al. dataset. UBE2S is over-expressed in the high risk group while SALL2 is under-expressed. This agrees with that UBE2S is a promoting breast cancer gene while SALL2 is a suppressor breast cancer gene [44, 46]. Additionally, UBE2S has higher scaled node degree in the high risk group while SALL2 has higher scaled node degree in the low risk group $\left (sd_{UBE2S}^{h}=0.286, sd_{UBE2S}^{l}=0.778, sd_{SALL2}^{h}=\right.\left.1.0, sd_{SALL2}^{l}=0.444\right)$. This shows, as a promoting breast cancer gene, UBE2S is more actively connected with its neighbors in the high risk group while, the suppressor breast cancer gene, SALL2 is more actively connected with its neighbors in the low risk group. In Fig. 7, yellow edges represent connections that have been supported from STRING database. We can see that these connections based on prior biological knowledge are not always showing up from the output of wgLASSO. This is a nice property since prior biological knowledge only provides evidence. We still need the support from the data to make a connection. Therefore, by integrating prior biological knowledge into data-driven models, we expect to build more robust and biologically relevant networks. Table 3 shows the survival time prediction performance when the top 5, top 10 and top 15 significant genes are selected by each of the four methods as the inputs to the multivariate Cox regression model (Fig. 6). In all three cases, the proposed dwgLASSO algorithm with prior biological knowledge incorporated achieved the best performance, followed by KDDN and dwgLASSO without prior biological knowledge incorporated. The method that relies purely on concordance index had the least performance.

**Table 3:** The survival time prediction performance (*p*-value and hazard ratio) for the top 5, top 10 and top 15 significant genes based on concordance index: DEA, dwgLASSO with no prior biological knowledge incorporated: dwgLASSO (no prior), KDDN, and dwgLASSO with prior biological knowledge incorporated: dwgLASSO (prior)

Methods	Top 5 significant genes		Top 10 significant genes		Top 15 significant genes	
	*p*-value	Hazard ratio	*p*-value	Hazard ratio	*p*-value	Hazard ratio
DEA	0.0073	1.851	2.00E-03	2.037	4.00E-04	2.274
dwgLASSO (no prior)	0.0066	1.864	3.10E-04	2.316	4.60E-06	2.969
KDDN	0.0022	2.028	7.46E-07	3.304	8.04E-06	2.889
dwgLASSO (prior)	**0** **.** **0** **0** **1** **3**	**2** **.** **1** **0** **4**	**7** **.** **0** **1** **E** **−** **0** **7**	**3** **.** **3** **2** **5**	**9** **.** **3** **7** **E** **−** **0** **7**	**3** **.** **2** **5**

The best performance is marked in bold when the gene number is fixed

### RNA-seq data

**Fig. 8:**
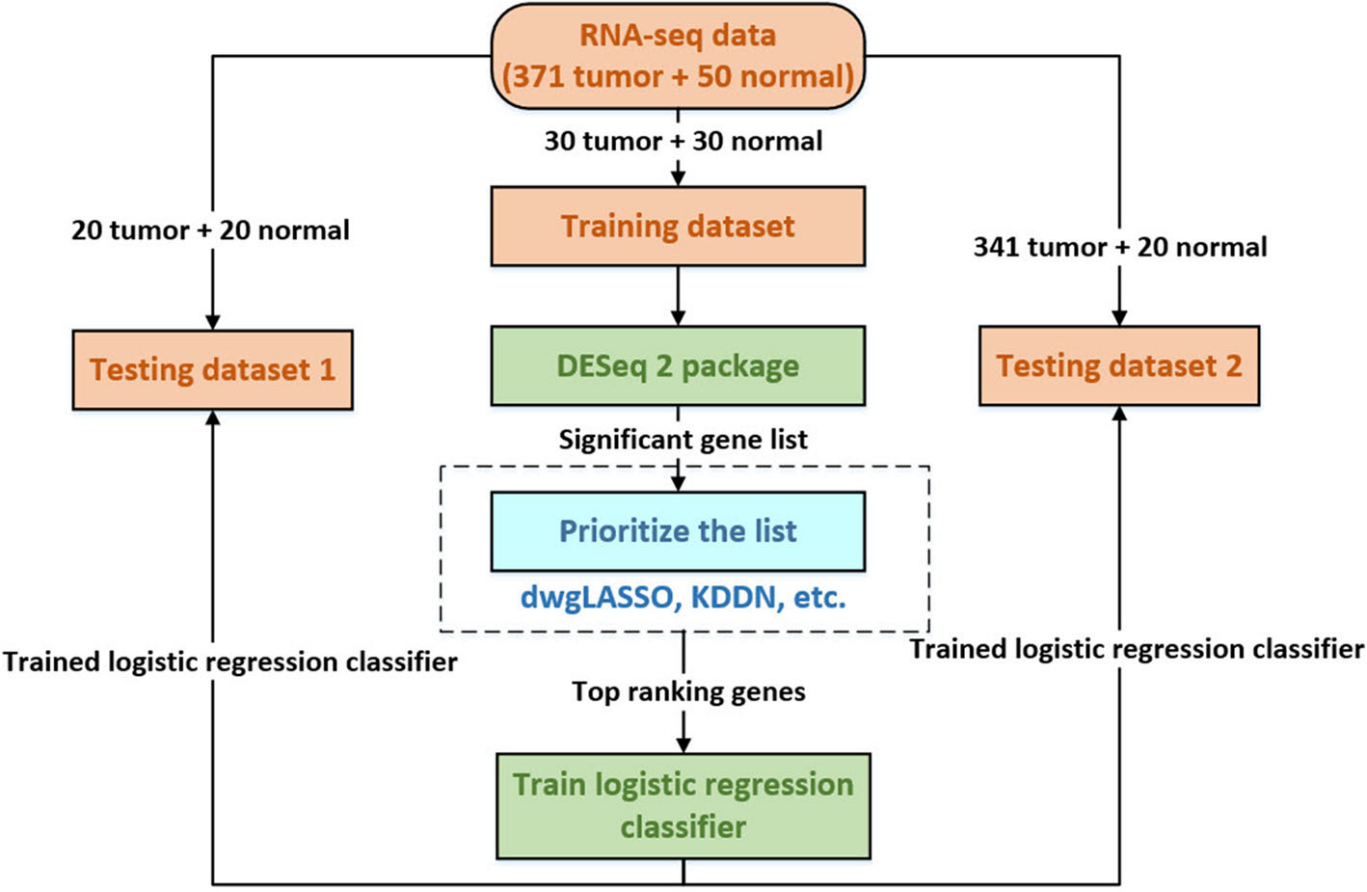
Workflow of dwgLASSO for more accurate classification prediction on RNA-seq data

Using UCSC Cancer Genomics Browser, we obtained TCGA RNA-seq data (level 3) acquired from patients with HCC [49]. The RNA-seq data was acquired by analysis of 423 liver tissues, including 371 primary tumor, 50 solid normal and 2 recurrent tumor samples based on Illumina HiSeq 2000 RNA Sequencing platform and mapped onto the human genome coordinates using UCSC cgData HUGO probeMap. Among the 371 primary tumor samples, 50 of them can find its corresponding solid normal samples. To evaluate dwgLASSO on RNA-seq data, we apply a workflow shown in Fig. 8. We first picked out the 100 samples whose tumor tissues and their corresponding non-tumorous tissues can both be found. Randomly, we selected 60 of them (30 tumor samples and their corresponding normal samples) as the training dataset. The remaining 40 samples (20 tumor samples and their corresponding normal samples) were used as testing dataset 1. Considering testing dataset 1 only contains 40 samples, we created testing dataset 2 by combining the above 40 samples and the remaining 321 tumor samples whose corresponding normal samples cannot be found. With testing datasets 1 and 2, we evaluated the performance of dwgLASSO on both balanced and large sample size datasets. Specifically, we preprocessed RNA-seq data using R package DESeq2 on the training dataset [50]. From DESeq2, we selected statistically significant genes whose adjusted *p*-values were less than 0.01 for subsequent analysis. At this step, the number of significant genes is typically between 1000 and 2000. We prioritized the significant gene list based on dwgLASSO. From the prioritized gene list, the top 5 genes were selected to train a logistic regression classifier to distinguish tumor and normal samples. The trained logistic regression classifier was finally evaluated on testing datasets 1 and 2. To compare dwgLASSO with other methods, we also prioritized the significant gene list based on adjusted *p*-value from DESeq2, dwgLASSO without prior biological knowledge incorporated and KDDN, built logistic regression classifier using the top 5 genes on the prioritized list and evaluated the trained classifier on the testing datasets 1 and 2.

The above procedure was repeated 100 times and the means and standard deviations for sensitivity, specificity and area under curve (AUC) were calculated using testing datasets 1 and 2 as shown in Table 4. In agreement with microarray data, network-based methods with prior biological knowledge incorporated yielded the best performance, followed by network-based method without prior biological knowledge incorporated, and the conventional differential gene expression analysis method was the worst. This is expected since both dwgLASSO and KDDN methods take into account of the changes of genes at gene expression and network topology levels, and incorporate prior biological knowledge into their network models.

**Table 4:** The mean and standard deviation (in parenthesis) of sensitivity, specificity and area under curve (AUC) calculated for conventional differential gene expression analysis: DEA, dwgLASSO with no prior biological knowledge incorporated: dwgLASSO (no prior), KDDN, and dwgLASSO with prior biological knowledge incorporated: dwgLASSO (prior)

Methods	Testing dataset 1			Testing dataset 2		
	Specificity	Sensitivity	AUC	Specificity	Sensitivity	AUC
DEA	0.950 (0.07)	0.913 (0.06)	0.951 (0.04)	0.950 (0.07)	0.941 (0.04)	0.983 (0.01)
dwgLASSO (no prior)	**0** **.** **9** **8** **8** **(** **0** **.** **0** **3** **)**	0.888 (0.11)	0.972 (0.02)	**0** **.** **9** **8** **8** **(** **0** **.** **0** **3** **)**	0.956 (0.05)	0.990 (0.01)
KDDN	0.963 (0.08)	**0** **.** **9** **5** **0** **(** **0** **.** **0** **4** **)**	0.980 (0.02)	0.963 (0.08)	0.939 (0.03)	0.989 (0.01)
dwgLASSO (prior)	**0** **.** **9** **8** **8** **(** **0** **.** **0** **3** **)**	0.950 (0.07)	**0** **.** **9** **8** **2** **(** **0** **.** **0** **3** **)**	**0** **.** **9** **8** **8** **(** **0** **.** **0** **3** **)**	**0** **.** **9** **6** **5** **(** **0** **.** **0** **3** **)**	**0** **.** **9** **9** **4** **(** **0** **.** **0** **1** **)**

The best performance is marked in bold

## Conclusion

In this paper, we apply a novel network inference method, wgLASSO to integrate prior biological knowledge into a data-driven model. We also propose a new network-based differential gene expression analysis method dwgLASSO for better identification of genes associated with biologically disparate groups. Simulation results show that wgLASSO can achieve better performance in building biologically relevant networks than purely data-driven models (e.g., neighbor selection and graphical LASSO) even when only a moderate level of information is available as prior biological knowledge. We demonstrate the performance of dwgLASSO in survival time prediction using two independent microarray breast cancer datasets previously published by Bild et al. and van de Vijver et al. The top 10 genes selected by dwgLASSO based on the dataset from Bild et al. dataset lead to a significantly improved survival time prediction on the dataset from van de Vijver et al., compared with the top 10 significant genes obtained by conventional differential gene expression analysis. Among the top 10 genes selected by dwgLASSO, UBE2S, SALL2, XBP1 and KIAA0922 have been previously reported to be relevant in breast cancer biomarker discovery study. We also tested dwgLASSO using TCGA RNA-seq data acquired from patients with HCC on tumors samples and their corresponding non-tumorous liver tissues. Improved sensitivity, specificity and AUC were observed when comparing dwgLASSO with conventional differential gene expression analysis method. Future research work will focus on applying dwgLASSO on other omic studies such as proteomics and metabolomics.

## Additional files

Additional file 1 **Table S1:** The total 58 significant genes along with their associated adjusted *p*-values. (CSV 1.09 kb)

Additional file 2 **Table S2:** The top 10 significant genes based on KDDN and dwgLASSO without prior biological knowledge incorporated. (CSV 4.00 kb)

## Abbreviations

AIC: Akaike information criterion
AUC: area under curve
BIC: Bayesian information criterion
DEA: differential gene expression analysis
DN: differential network
dwgLASSO: differentially weighted graphical LASSO
FDR: false discovery rate
FP: false positives
FN: false negatives
GGMs: Gaussian graphical models
HCC: hepatocellular carcinoma
KDDN: Knowledge-fused differential dependency network
LASSO: least absolute shrinkage and selectioin operator
MAP: maximum a posteriori
MLE: maximum likelihood estimation
PPI: protein-protein interaction
wgLASSO: weighted graphical LASSO
